# Tactile-dependant corticomotor facilitation is influenced by discrimination performance in seniors

**DOI:** 10.1186/1744-9081-6-16

**Published:** 2010-03-05

**Authors:** Sabah Master, François Tremblay

**Affiliations:** 1 School of Psychology University of Ottawa, Ottawa, Ontario, K1N 6N5, Canada; 2 School of Rehabilitation Sciences, University of Ottawa, Ottawa, Ontario, K1H 8 M5, Canada

## Abstract

**Background:**

Active contraction leads to facilitation of motor responses evoked by transcranial magnetic stimulation (TMS). In small hand muscles, motor facilitation is known to be also influenced by the nature of the task. Recently, we showed that corticomotor facilitation was selectively enhanced when young participants actively discriminated tactile symbols with the tip of their index or little finger. This tactile-dependant motor facilitation reflected, for the large part, attentional influences associated with performing tactile discrimination, since execution of a concomitant distraction task abolished facilitation. In the present report, we extend these observations to examine the influence of age on the ability to produce extra motor facilitation when the hand is used for sensory exploration.

**Methods:**

Corticomotor excitability was tested in 16 healthy seniors (58-83 years) while they actively moved their right index finger over a surface under two task conditions. In the tactile discrimination (TD) condition, participants attended to the spatial location of two tactile symbols on the explored surface, while in the non discrimination (ND) condition, participants simply moved their finger over a blank surface. Changes in amplitude, in latency and in the silent period (SP) duration were measured from recordings of motor evoked potentials (MEP) in the right first dorsal interosseous muscle in response to TMS of the left motor cortex.

**Results:**

Healthy seniors exhibited widely varying levels of performance with the TD task, older age being associated with lower accuracy and vice-versa. Large inter-individual variations were also observed in terms of tactile-specific corticomotor facilitation. Regrouping seniors into higher (n = 6) and lower performance groups (n = 10) revealed a significant task by performance interaction. This latter interaction reflected differences between higher and lower performance groups; tactile-related facilitation being observed mainly in the former group. Latency measurements and SP durations were not affected by task conditions.

**Conclusions:**

The present findings provide further insights into the factors influencing task-dependant changes in corticomotor excitability in the context of aging. Our results, in particular, highlight the importance of adjusting task demands and controlling for attention when attempting to elicit task-specific motor facilitation in older persons engaged in fine manual actions. Such information could be critical in the future for planning interventions to re-educate or maintain hand function in the presence of neurological impairments.

## Background

In everyday life, we often rely on our sense of touch when it comes to appreciating object and surface properties, as when searching for keys inside the pocket. Such a task typically engages the finger in fine exploratory movements to detect specific tactile features, which can then lead to fast object recognition [1]. While trivial in appearance, tactile discrimination (TD) tasks have been shown to engage a large cortical network involving primary and secondary motor and sensory areas, as well as, associative regions of the frontal and parietal lobes [2-5]. Recently [6], we investigated task-dependant changes in corticomotor excitability with transcranial magnetic stimulation (TMS) when young adults actively moved their index or little finger over a surface. Our results showed a large selective enhancement in corticomotor excitability when participants discriminated between surface features, as opposed to simply moving the finger over a blank surface. Further to this, we showed that such tactile-dependant extra facilitation was largely abolished when participants performed a concurrent distraction task. This suggested that task influences, linked with the increased attentional demands associated with tactile sensing, were primarily responsible for the observed extra facilitation.

In the present report, we attempted to extend those observations on tactile-dependant increase in corticomotor excitability to healthy seniors to investigate whether age-related alterations in sensorimotor capacities and cognitive functions would affect the ability to produce motor facilitation in the context of active finger movements. Previous reports in this regard have produced mixed results. For instance, D'Esposito et al. [7] examined task-dependant changes in motor cortical activation using functional magnetic resonance imaging (fMRI) and reported an age-related decrease both in the number of subjects showing detectable activation and in the volume of activation during performance of a button press task. This suggested a decline with age in the ability to activate the motor cortex during simple finger movement execution. The fact that older subjects also displayed higher levels of background noise might have affected the results, however. The issue of age-related differences in motor activation was further examined by McConnell et al. [8] who combined TMS with fMRI to measure motor cortical activity induced either by volitional movement or by direct stimulation using TMS. Their results revealed no differences in haemodynamic responses with age for both voluntary-induced and TMS-induced finger movements, indicating a preserved capacity to drive the corticomotor system in normal aging. Similar results were reported by Sale and Semmler [9] who examined right-left differences in corticomotor excitability in young and old adults. Their results showed that older adults had preserved MEP responses in the right hand, although MEP amplitude tended to be reduced in the left hand. No such bilateral difference was found in young adults. Interestingly, they observed that performance of a complex hand action (using gardening shears, as opposed to a simple action) produced large MEP facilitation in the right hand of old adults, whereas no such facilitation could be elicited in the left hand. The authors attributed this asymmetry in the older group to a lifetime preferential use of the dominant hand in executing fine motor tasks.

Using a different task paradigm, Leonard and Tremblay [10] showed recently that older adults were capable of producing corticomotor facilitation in their dominant hand in the context of overt and covert action execution, i.e. during observation, imagination and imitation of a complex hand action (scissoring action). This preserved capacity for motor facilitation in older adults was however less specific than that seen in young adults in terms of muscle selectivity; older adults showing facilitation in both the task and non-task relevant muscles. This loss in selectivity was thought to reflect compensatory mechanisms in older adults whereby performance of simple motor actions often leads to extra activation in areas that are not normally recruited in younger subjects, such as the pre-supplementary motor area, pre-dorsal premotor area, rostral cingulate, and prefrontal cortex [11-14]. For Heuninckx et al. [11], such a wide activation extending to associative areas of the cortex reflected the need for older adults to exert greater cognitive control over on-going actions to maintain performance at the desired level [see also [15]]. This penetration of cognition into motor control with age raises the issue of resource allocation when task demands are increased, given the expected decline in selective attention and working memory with aging [16,17]. With limited resources, older participants might be particularly challenged when hand movements require fine control such as when the finger is used to explore tactile features for recognition. As stated earlier, in the present report, we attempted to address this issue using TMS to measure task-dependant corticomotor facilitation elicited in a demanding task paradigm wherein participants had to actively move their index finger at a prescribed speed over a surface with or without constraints for tactile sensing at the fingertip.

## Methods

The Institutional Review Ethics Board approved the study procedure in accordance with the principles of the Declaration of Helsinki and informed consent was obtained before the experimental session. All assessments were performed in a controlled laboratory environment. Each participant received an honorarium for his or her participation.

The methods and procedures have been detailed previously in our report [see [6]] in young adults, where we used the same experimental paradigm as in the present experiment. Briefly, corticomotor facilitation was tested in a group of healthy seniors (8 female, 8 male; mean age: 68.0 years, range: 58-83 years, 15 right-handers) using a Magstim 200 stimulator (Magstim Co. Dyfed, UK) connected to a figure-eight coil (70 mm loop diameter). Before testing participants were screened for contra-indications to TMS and for the presence of sensory neuropathies using a graduated Rydel-Seiffer tuning fork [18,19]. Corticomotor excitability was determined by monitoring changes in the amplitude and latency of motor evoked potentials recorded in the first dorsal interosseous (FDI) using surface electrodes (10 mm diameter, Ag-AgCl). We intended initially, as in our previous study in young adults, to include observations on *adductor digiti minimi*, but this turned out to be impossible because most participants could not perform the task properly with the little finger. Electromyographic (EMG) signals were amplified and filtered (5 Hz to 5 kHz) using a polygraph amplifier (RMP-6004, Nihon-Kohden Corp.) and stored on computer (digitized @ 1 kHz, BNC-2090, National Instrument Corp.) for off-line analyses.

**Figure 1:**
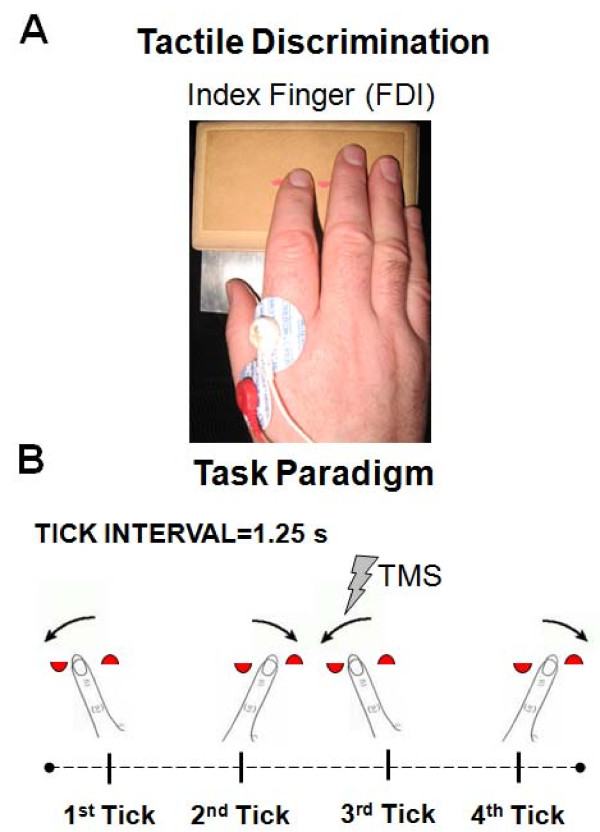
**Experimental set-up and schematic illustration of the TD paradigm**. A. Experimental set-up showing the position of the index finger just before TMS pulse delivery during performance of the tactile discrimination (TD) task and the location of the recording surface electrodes. B. Schematic illustration of the task paradigm used to assess motor evoked potential (MEP) facilitation. In both the TD and non discrimination (ND) tasks, participants were trained to produce rhythmic finger to and fro movements in sync with the sound of a metronome at a frequency of 0.8 Hz for 5 s. In the TD condition, the finger moved over a surface containing two tactile symbols (half circle stickers forming either a "Boat" or a "Hat", 3.2 mm radius, 16 mm apart) and participants were asked to report the location of one of the specified symbols at the end of the trial. The ND task was identical in performance to the TD task except that the finger moved over a blank surface. In each trial, the TMS pulse was set to trigger at a time corresponding to the 3rd tick (3.75 s) in the trial when the index finger was moving towards abduction.

Testing of task-related corticomotor facilitation was performed with participants blindfolded and seated in a recording chair. Prior to testing, participants underwent a period of familiarization with the task conditions. During that period, they were trained to produce rhythmic index finger to and fro movements in sync with metronome ticks at 0.8 Hz for 5 s. Then, participants were introduced to the two task conditions: 1) no discrimination (ND) and 2) tactile discrimination (TD). In the ND condition, participants simply moved their finger in sync with the ticks on a blank wooden block. In the TD condition, participants were required as they moved their finger to attend to the position of two tactile symbols (hat and boat) formed by two half-circle stickers (3.2 mm radius, 16 mm apart) pasted on a wooden block (see Figure 1). Participants had to report the location (right or left) of one of the tactile symbols in each trial within a block of 16 trials. Once familiarized with the task conditions, corticomotor excitability was tested in each participant under the two task conditions (TD and ND), the order of testing in the two being counterbalanced across participants. Corticomotor excitability was tested during each task by delivering a TMS pulse at 110% of the relaxed motor threshold at 3.75 s in the course of the task execution. This timing was selected based on our previous report in young adults and corresponded to the 3rd tick in the sequence of cyclic movements when the FDI was actively moving the index finger into abduction (see Figure 1). Sixteen trials of 1000 ms epochs were recorded under each task condition. MEP amplitude, latency, and EMG traces were measured off-line and averaged to derive mean individual values. The EMG activity in the 500 ms preceding the TMS pulse was rectified, averaged and expressed as a percentage of the maximum voluntary contraction. Finally, the silent period (SP) was estimated as the interval from MEP onset to the first sign of EMG return.

A paired-samples t-test was performed on background EMG levels (% MVC) recorded during the two tasks, TD and ND. As suggested by Nielsen [20] and Schmidt et al. [21], MEP amplitudes were log-transformed to get a normal distribution (Shapiro-Wilk P > 0.1). Repeated measures analyses of variance (ANOVAs) were then performed on the dependent variables of MEP log-amplitude, MEP latency, and SP duration with task condition (TD, ND) as the repeated factor and discrimination performance as the between-subjects factor. The latter factor was entered into the ANOVA as a dichotomized variable (high vs. low) after examining the distribution of individual performance values with respect to age (see Results). The level of significance was set at P < 0.05. All tests were performed using SPSS software version 17.0 for Windows™ (Chicago, IL, USA). Figures were prepared using GraphPad Prism version 5.02 for Windows (GraphPad Software, San Diego California USA, http://www.graphpad.com). All values are reported as mean ± 1 SD.

## Results

### Task performance

**Figure 2:**
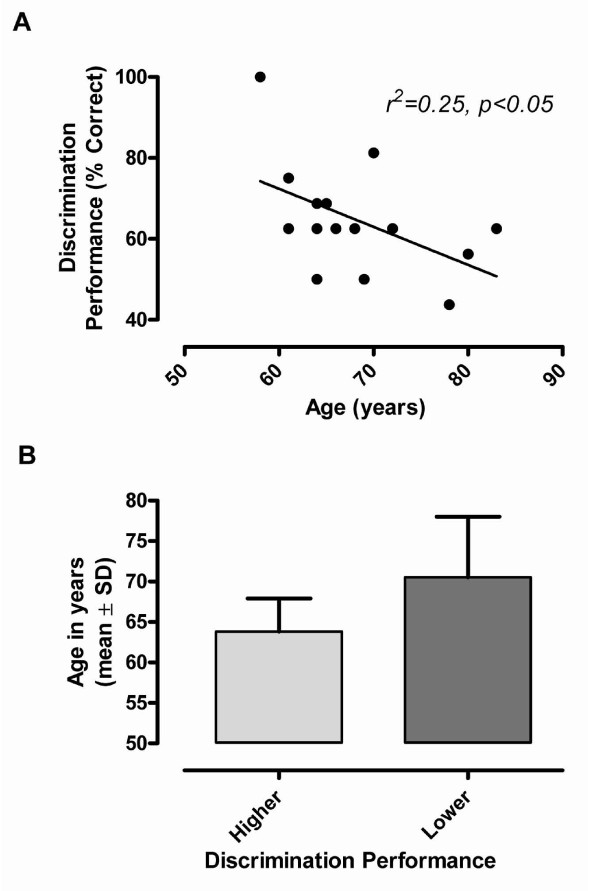
**Age and task performance**. A. Scatterplot showing the inverse association between performance in the TD task and age (r value represents Pearson's correlation coefficient). B. Comparison of the mean age of participants after allocation to either a higher performance (≥ 69% correct, n = 6) or a lower performance group (<63% correct, n = 10). Note that individuals with higher discrimination performance were significantly younger (64 ± 4.1 yrs) than those (71 ± 7.5) with lower performance (t_14 _= 1.99, p = 0.03).

Participants exhibited various levels of performance in discriminating between the two symbols while performing the TD task (i.e., from perfection down to chance level; mean correct 65 ± 13%). Further inspection of individual performance data indicated that the older seniors generally experienced greater difficulty in performing the task than younger seniors. This inverse association is clearly apparent in Figure 2A, where corresponding individual performance data have been plotted with respect to age. Pearson's correlation confirmed that the two factors were indeed inversely related. On the basis of this relationship and given the difficult nature of the TD task, participants were regrouped into two subsets, i.e. those with a relatively high performance (≥ 69% correct, mean 77 ± 12%) and those with a relatively low performance (< 63% correct, mean 58 ± 7%). As evident in Figure 2B, the two subsets also reflected the age difference, the lower performance group being, on average, seven years older (71 ± 8 yrs) than the higher performance group (64 ± 4 yrs; t_14 _= 1.99, p = 0.03).

**Figure 3:**
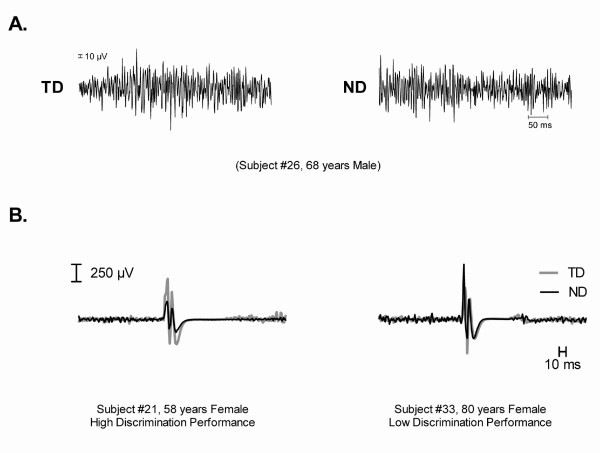
**Electromyographic (EMG) activity and MEP traces**. A. typical example of electromyographic (EMG) activity elicited in the FDI muscle during performance of the two finger movement tasks (discrimination, TD; non discrimination, ND). Note the similarity between the two tasks (TD vs. ND) in terms of EMG patterns produced. B. Examples of task-specific facilitation recorded in the FDI in two individuals, one having high (100%) and another having low (56%) discrimination performance. Note the task-related increase in MEP amplitude in the TD condition in the younger senior with high discrimination performance, and the similarity in MEP amplitudes between the TD and ND conditions in the older senior with low discrimination performance.

While the TD task proved to be challenging perceptually for seniors, the performance of the task at the motor level was not different from that seen when executing the ND task. A typical example of EMG activity recorded in both task conditions is shown in Figure 3A. In terms of background muscle activation, the two tasks elicited relatively low levels of EMG activity in the FDI (mean TD, 11.8%; ND, 14.8% of the MVC). As in our previous study using the TD paradigm in young adults [6], no difference in EMG levels was found between the two tasks (t_15 _= -1.09, p = 0.29).

### Task-specific facilitation

**Figure 4:**
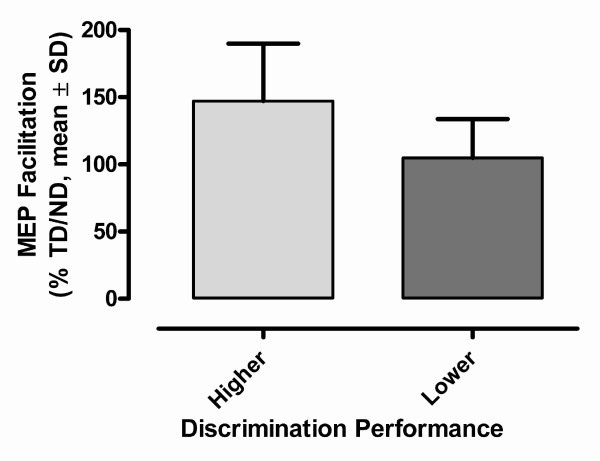
**Tactile-related MEP increase and performance level**. Mean tactile-related changes in MEP amplitude are shown for the two subsets of participants, i.e. higher (n = 6) and lower (n = 10) performance groups. Each bar represents the average of individual changes in MEP amplitude measured under the TD task using the ND task as a baseline (i.e., %TD/ND).

As observed for the discrimination performance, senior participants also exhibited a great deal of variability in terms of modulation of MEP amplitude in response to changes in task conditions (mean relative increase, 21 ± 39%). In spite of this variability, a significant main effect of task conditions on MEP amplitude was detected in the ANOVA (F_1,14 _= 5.35, p = 0.04). The ANOVA also revealed a significant task × performance interaction (F_1,13 _= 5.82, p = 0.03), which indicated that performance levels (high vs. low) did influence observed task-related variations in MEP amplitude. In fact, this interaction accounted for 26% of the overall variance in MEP amplitude. Typical examples of MEP modulation seen under the two task conditions are illustrated in Figure 3B, where the contrast between high and low performance is easily apparent. The influence of discrimination performance on MEP amplitude can also be appreciated in Figure 4, which compares the mean tactile-related change in MEP amplitude (TD/ND) in the two performance groups. Latency (TD, 20.2 ± 2.2 ms; ND, 20.1 ± 1.6 ms) and SP durations (TD, 70 ± 29 ms, ND, 77 ± 30 ms) did not differ between task conditions, and no interaction was found with discrimination performance (F_1,14 _< 1, p > 0.1).

## Discussion

In the present report, we extend our previous observations on task-specific motor facilitation in young adults [6] to healthy seniors. In this respect, our results reveal important differences between our former observations in young adults and the way seniors responded to increasing task demands in the context of this experiment. First, seniors tended to show much larger variability in terms of their ability to cope with increasing task demands, i.e. from simple auditory paced rhythmic finger movements to rhythmic finger movement combined with tactile sensing. Second, age greatly influenced task performance, which in turn, affected levels of MEP facilitation.

The issue of greater variability in behavioural and neurophysiological responses with advancing age is a common theme in aging studies. In the present study, participants in the older age group tended to exhibit lower performance with accompanying low or absent MEP facilitation under the TD condition, when compared to younger seniors. In our previous work on tactile sensation and aging, we observed a similar pattern of results with more variable performance and greater decline in tactile acuity being observed for individuals over 75 years [22,23]. Such variability was also observed in a TMS study by Peinemann et al. [24] looking at levels of intra-cortical inhibition (ICI) and intra-cortical facilitation (ICF) elicited in older adults in response to paired pulse stimulation. They noticed, much like in the present study, a differential pattern of modulation in a subgroup of older participants aged >60 years, where the expected increase in ICF was actually replaced by a decline. An increase in MEP amplitude variability with age was also reported by Pitcher et al [25], when examining variations in MEP size with increasing TMS intensities (i.e., stimulus-response curve). Interestingly, they found that the age-related difference in stimulus-response profiles, reflecting the strength of corticospinal projections, was evident only in older female subjects but not in male subjects; again illustrating the inherent variability associated with aging. Thus, it is not uncommon in aging studies to find subsets of participants exhibiting different patterns of responses, as we found in the present study.

Before addressing the issue as to why certain participants showed task-specific facilitation, while others did not, it is important to ascertain that motor over-activity was not a factor in limiting the ability to produce corticomotor facilitation. This question is critical since seniors tend to show compensatory activity and higher activation levels in both motor and non motor areas of the cortex even when executing simple finger movements [11-14,26,27]. This possibility is unlikely, however, given that no participants actually showed signs of MEP saturation under the TD task condition. In fact, MEP's were actually reduced in amplitude in all but two of the participants who failed to show extra facilitation with tactile sensing. The fact that the finger movements in the two tasks were associated with relatively low levels of background EMG activity likely contributed to limit the level of motor activation, associated with the finger movements. In fact, our observations of task-related MEP facilitation with tactile sensing in seniors fit with the recent findings of Van Impe et al. [28] who measured cortical activation during a hand-foot coordination task in older adults. Their results showed that, although age was associated with activation of a larger brain network, this activation reflected increased attentional deployment to enhance somatosensory processing and integration rather than increased motor cortical activity. Thus, other factors, besides motor over-activity, likely contributed to the variations observed in MEP amplitude under the two task conditions in our group of seniors.

We have already mentioned that performance in the tactile task largely influenced MEP facilitation in our group of seniors. In fact, the present results show that the degree of task-specific facilitation was linked with the actual perceptual performance of seniors in discriminating the tactile symbols; a higher performance being associated with large MEP facilitation, while a lower performance was not. In many respects, the present results are reminiscent of our previous findings in young adults, where high performance (mean, 84% correct) was associated with substantial tactile-related MEP facilitation (mean relative increase, 45%), while the same facilitation was abolished when attention was diverted away from the tactile inputs by performance of a concurrent cognitive task. Together, these observations strongly suggest that the observed task-related corticomotor facilitation seen during tactile sensing is central in origin, reflecting enhanced excitability mediated by top-down attentional mechanisms acting on the motor cortex to facilitate task performance. This idea is further supported by recent findings showing a participation of anterior motor cortical area 4 in complex somatosensory processing [29], highlighting the importance of finely tuned central motor control during the execution of tactile exploratory tasks.

In light of these observations, the inverse association between age and task performance can be explained, in the case of older seniors, by a difficulty in attending to the tactile stimuli as the finger moved over the surface. The converse can be said for younger seniors, where effective coping with task demands likely allowed them to selectively attend to the tactile symbols as the index finger moved, resulting in higher discrimination performance and MEP facilitation during tactile sensing. However, it is still possible that a greater degree of peripheral decline in tactile sensibility in older seniors might have affected their ability to sense the tactile spatial features when touching the stimuli. Two arguments mitigate this possibility, however. First, all participants were screened for the presence of sensory deficits at the outset of the study using validated vibratory thresholds as an index of tactile sensation. Second, the spatial dimensions of the tactile symbols (3.2 mm radius, 0.18 mm relief) were in the range of easily detectable spatial stimuli, even for individuals advanced in age [23]. In fact, the great majority of participants experienced no difficulties in discriminating between the two tactile symbols in the familiarization period before formal testing. It seems more likely, as we suggested above, that ineffective coping mechanisms in the context of multiple task demands was responsible for the poor performance in older seniors. Such an explanation would be consistent with observations suggesting that deficits in top-down modulation mechanisms are critical in leading to cognitive decline in normal aging; older adults being particularly impaired in their ability to selectively suppress task-irrelevant information [30]. As recently shown by Gazzeley et al [31], such an inability seems to result from excessive attention towards distracting stimuli early in the sensory encoding process, resulting in lower processing speed and decreased performance (i.e., longer response time and lower accuracy). In the context of our TD task, the sound of metronome ticks in the background could have drawn too much attention on the part of certain older seniors to the detriment of the tactile information arising from contact with the symbols; leading to low discrimination performance and inefficient task-related corticomotor facilitation.

The deficit in top-down modulation with age is thought to correspond to changes in the frontal and parietal lobes and the resulting decreased connectivity of the anterior-to-posterior network, in particular the frontal associative areas and the motor cortex [32,33]. In support of this argument, Rowe [27] showed recently that cortical connectivity between the contralateral premotor and prefrontal cortices was impaired during an externally paced randomized button-pressing task in seniors. Indeed, the anterior-to-posterior network would have been important for the TD task, given that it is involved in haptic sensing [34-36] and attention to action [37]. A recent review of the literature on somatosensory-motor interactions by Bressler [38] supports the idea that attentional mechanisms are part of the large-scale, synchronized cortical network controlling motor activity, and can mediate the critical relationship between the somatosensory and motor cortices.

## Limitations

The present results are based on a relatively small sample of healthy seniors, which might not be representative of the elderly population in general. In addition, the degree of difficulty associated with the TD task proved to be very challenging for some seniors. It would be important for future studies investigating task-related motor facilitation in older adults to control for the degree of task difficulty, providing some adjustments when necessary, to account for the increased variability generally observed in this population in terms of perceptual performance.

## Conclusions

In conclusion, the present findings provide further insights into the factors influencing tactile-dependant changes in corticomotor excitability, in the context of aging. Our results, in particular, highlight the importance of adjusting task demands and modulating attentional influences at the individual level to elicit proper task-specific facilitation when older persons are engaged in fine motor actions. Such information could be critical in the future for planning interventions to re-educate hand function in the presence of neurological impairments.

## Competing interests

The authors declare that they have no competing interests.

## Authors' contributions

SM participated in the design of the study, carried out the behavioral testing, performed the statistical analysis and drafted the manuscript. FT conceived of the study, and participated in its design and coordination and drafted the manuscript. Both authors read and approved the final manuscript.
